# A Method for Real-Time Assessment of Mitochondrial Respiration Using Murine Corneal Biopsy

**DOI:** 10.1167/iovs.64.11.33

**Published:** 2023-08-29

**Authors:** Wentao Liang, Li Huang, Tian Yuan, Rui Cheng, Yusuke Takahashi, Gennadiy P. Moiseyev, Dimitrios Karamichos, Jian-Xing Ma

**Affiliations:** 1 Department of Physiology, University of Oklahoma Health Sciences Center, Oklahoma City, Oklahoma, United States; 2 Department of Ophthalmology, Fujian Medical University Union Hospital, Fuzhou, China; 3 Department of Biochemistry, Wake Forest University School of Medicine, Winston-Salem, North Carolina, United States; 4 North Texas Eye Research Institute, University of North Texas Health Science Center, Fort Worth, Texas, United States; 5 Department of Pharmaceutical Sciences, University of North Texas Health Science Center, Fort Worth, Texas, United States; 6 Department of Pharmacology and Neuroscience, University of North Texas Health Science Center, Fort Worth, Texas, United States

**Keywords:** cornea, mitochondria, metabolism, biopsy, Seahorse

## Abstract

**Purpose:**

To develop and optimize a method to monitor real-time mitochondrial function by measuring the oxygen consumption rate (OCR) in murine corneal biopsy punches with a Seahorse extracellular flux analyzer.

**Methods:**

Murine corneal biopsies were obtained using a biopsy punch immediately after euthanasia. The corneal metabolic profile was assessed using a Seahorse XFe96 pro analyzer, and mitochondrial respiration was analyzed with specific settings.

**Results:**

Real-time adenosine triphosphate rate assay showed that mitochondrial oxidative phosphorylation is a major source of adenosine triphosphate production in ex vivo live murine corneal biopsies. Euthanasia methods (carbon dioxide asphyxiation vs. overdosing on anesthetic drugs) did not affect corneal OCR values. Mouse corneal biopsy punches in 1.5-mm diameter generated higher and more reproducible OCR values than those in 1.0-mm diameter. The biopsy punches from the central and off-central cornea did not show significant differences in OCR values. There was no difference in OCR reading by the tissue orientations (the epithelium side up vs. the endothelium side up). No significant differences were found in corneal OCR levels between sexes, strains (C57BL/6J vs. BALB/cJ), or ages (4, 8, and 32 weeks). Using this method, we showed that the wound healing process in the mouse cornea affected mitochondrial activity.

**Conclusions:**

The present study validated a new strategy to measure real-time mitochondrial function in fresh mouse corneal tissues. This procedure should be helpful for studies of the ex vivo live corneal metabolism in response to genetic manipulations, disease conditions, or pharmacological treatments in mouse models.

Dysregulated mitochondrial metabolism is imperative in various pathologies, such as metabolic diseases, trauma, and aging.^1^^–^^4^ Mitochondrial oxygen consumption in freshly isolated mitochondria, cultured cells, or tissues is commonly assessed using a Seahorse Extracellular Flux Analyzer.^5^^–^^7^ However, the mitochondrial isolation procedure may alter organelle functions by disrupting the cell/organelle structures and intracellular interactions.^8^ Moreover, in vitro measurement using cultured cells could not adequately reflect the in vivo status, because tissular/cellular microenvironments and the cell-to-cell interactions may be altered. To overcome this limitation, assessing mitochondrial function in fresh tissues is of great interest.

Growing evidence has shown that disturbed metabolism plays a key role in the pathophysiology of corneal diseases.^9^^–^^11^ However, the direct impacts of different disease conditions on corneal mitochondrial metabolism are not fully investigated owing to limited methods for measuring metabolism in ex vivo live corneal tissue. The present study aimed to develop an assay for the real-time measurement of mitochondrial metabolism in ex vivo live murine corneas.

To investigate the mitochondrial function in ex vivo live cornea tissues, we developed and validated a new method using a Seahorse XFe96 pro Extracellular Flux Analyzer. We evaluated the impacts of different euthanasia methods, punch sizes, and biopsy locations on oxygen consumption rate (OCR). In addition, we compared the corneal OCR values in different sexes, ages, and strains. Using this novel method, we found that diabetes impaired corneal mitochondrial function in type 1 and type 2 diabetic mouse models.^12^ Altogether, these results will greatly facilitate the study of real-time mitochondrial function in ex vivo live murine cornea.

## Methods

### Animals

C57BL/6J (#000664) and BALB/cJ (#000651) mice were purchased from Jackson Laboratories (Bar Harbor, ME, USA). All experiments were in adherence to the ARVO Statement for the Use of Animals in Ophthalmic and Vision Research and approved by the Institutional Animal Care and Use Committee of Wake Forest University School of Medicine.

### Corneal Biopsy Preparation

**Figure 1.**
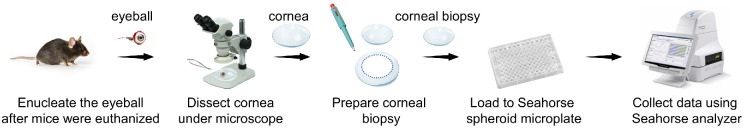
Overall workflow of the research procedure. Corneal biopsies were obtained using disposable biopsy punches after euthanizing the mice. The biopsies were loaded into a Seahorse XFe96 spheroid microplate, one per well. The metabolic profile and mitochondrial respiration rates of the biopsies were assessed using a Seahorse XFe96 pro analyzer.

The mice were euthanized either by carbon dioxide asphyxiation controlled by a carbon dioxide regulator flowmeter (Western Enterprises #M1-320-12FMH, Westlake, OH, USA) or an overdose of anesthetic drug, which is a combination of 500 mg/kg ketamine (VETONE #13985-584-10, Boise, ID, USA) and 50 mg/kg xylazine (Bimeda #200-529, Le Sueur, MN, USA) supplement administered via intraperitoneal injection. Subsequently, the whole corneas were obtained after euthanization (Fig. 1). Corneal biopsies prepared using a disposable biopsy punch in 1.0-mm diameter (VWR #33-31AA, Integra LifeSciences, Princeton, NJ, USA) or 1.5-mm diameter (VWR #33-31A) were loaded into a Seahorse XFe96 spheroid microplate (Agilent Technologies, 102978-100, Wilmington, DE, USA) one biopsy per well. Corneal biopsy punches were kept in Seahorse XF base medium (Agilent 103335-100) containing 10.0 mM glucose (Sigma #G7528, St. Louis, MO, USA), 1.0 mM pyruvate (Sigma #S8636), and 2.0 mM L-glutamine (Sigma #G7513) supplement on ice until measurement.

### Real-Time Adenosine Triphosphate (ATP) Rate Assay

A Seahorse XF real-time ATP rate assay on the corneal biopsy was performed using the Agilent Seahorse XFe96 pro Analyzer. The basal OCR was measured first without any added compounds. Then, the OCR was recorded following the injection of oligomycin (Sigma #O4876) at a final concentration of 1.5 µM and a combination of rotenone (Sigma #R8875) and antimycin A (Sigma #A8674) (RAA) at a final concentration of 0.5 µM. For each step (basal, oligomycin, RAA), the measurement was repeated three times to obtain an average value. Each measurement was conducted for a duration of 3 minutes after a 3-minute mixing process, using a sensor cartridge to detect proton production and oxygen levels in each well. The Agilent Seahorse Wave Pro software (version 10.0.1) and the Real-Time ATP Rate Assay Report Generator were used for the calculation of ATP production according to the manufacturer's instructions. For detailed information, please refer to the related White Paper. Briefly, the assay initially measured the basal OCR and extracellular acidification rate. Subsequently, oligomycin was injected to inhibit ATP synthase (complex V), thereby blocking mitochondrial ATP production. Then, rotenone (complex I inhibitor) and antimycin A (complex III inhibitor) were used to assess nonmitochondrial respiration, primarily derived from glycolysis. By combining the extracellular acidification rate data with the cell medium buffering capacity factor, the software calculated the total, glycolytic, and mitochondrial ATP production.

### Mitochondrial Stress Test

The mitochondrial stress test was carried out following Agilent's protocol. The initial measurement was taken as the baseline, and no additional compounds were added during this step. Then, OCR was measured with sequential injections of 1.5 µM oligomycin, 2.0 µM (carbonyl cyanide 4-(trifluoromethoxy) phenylhydrazone (FCCP), (Sigma #C2920)), and 0.5 µM RAA. We carried out three repetitions of measurements to obtain average values for each stage (baseline, oligomycin, FCCP, RAA). Each measurement involved a 3-minute mixing phase followed by a 3-minute measurement period. The mitochondrial respiration was analyzed using Agilent Seahorse Wave Pro software and Mito Stress Test Report Generator.

### Corneal Epithelial Abrasion

Before the corneal punch procedure, the corneal epithelial wound was induced following a documented protocol.^13^ Briefly, an ocular burr (Alloy Medical, San Mateo, CA, USA) was used to generate an abrasion on the central cornea after mice were anesthetized by an intraperitoneal injection of 50 mg/kg ketamine hydrochloride mixed with 5 mg/kg xylazine.

### Statistical Analysis

The data were expressed as the mean ± standard deviation and analyzed using Prism 9 (GraphPad Software, Boston, MA, USA). The unpaired Student *t*-test was used to compare two groups, while a one-way analysis of variance was used for comparing more than two groups.

## Results

### Metabolic Profile of Murine Corneal Biopsy

**Figure 2.**
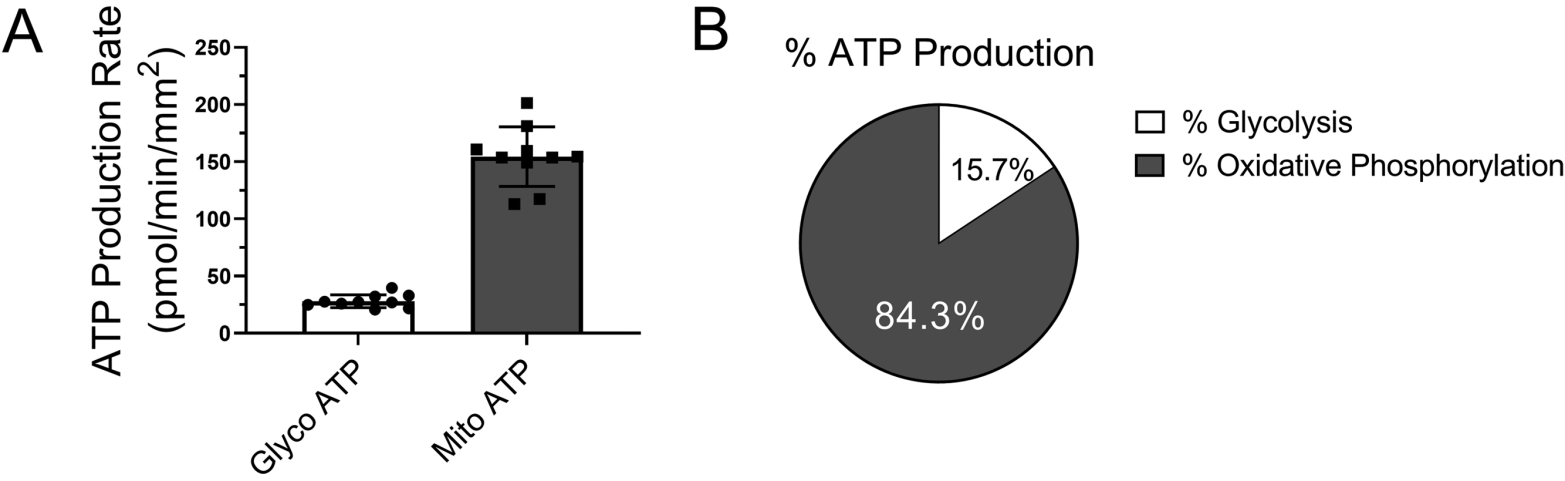
Metabolic profile of murine corneal biopsy. (**A**) ATP production from glycolysis (Glyco) and mitochondria (Mito) were measured in 1.5-mm diameter corneal punches with the epithelium side up from 8-week-old male C57BL/6J mice (*n* = 10 corneal biopsies from 5 mice) using real-time ATP rate assay. Values are expressed as mean ± standard deviation. (**B**) The average percentages of ATP production from glycolysis and oxidative phosphorylation in murine corneal biopsy (*n* = 10 corneal biopsies from 5 mice).

To study the metabolic profile of murine cornea, corneal biopsies in 1.5-mm diameter were used to evaluate the real-time ATP production with the epithelium side up by a Seahorse XFe96 pro Analyzer. Figures 2A and B showed that in the freshly isolated adult murine corneal biopsy, 84.3 ± 4.5% of total ATP was produced by oxidative phosphorylation; only 15.7 ± 4.5% was from glycolysis. This result suggested that mitochondrial oxidation is the major metabolism form in the cornea.

### Different Euthanasia Methods did not Affect OCR in the Murine Cornea

**Figure 3.**
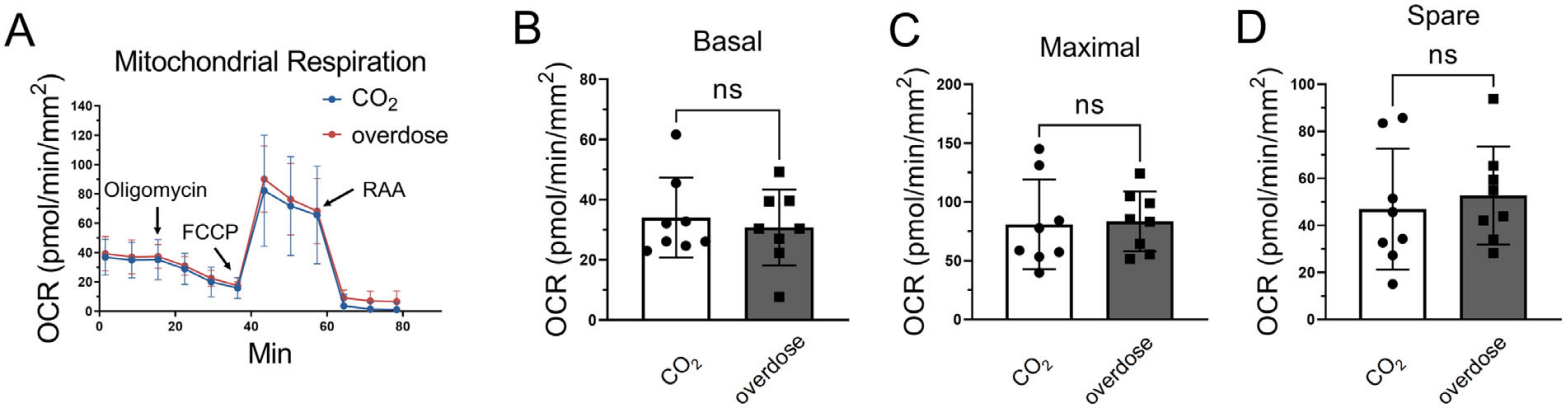
The impact of different euthanasia methods on mouse corneal oxygen consumption. (**A**) Mitochondrial stress test in the corneal biopsies from male C57BL/6J mice euthanized by carbon dioxide asphyxiation (CO_2_) and an overdose of the anesthetic drug (overdose). (**B**–**D**) Statistic analysis graphs of basal, maximal, and spare mitochondrial respiration capacities. Values are expressed as mean ± standard deviation, *n* = 8 corneal biopsies from 4 mice. FCCP, carbonyl cyanide 4-(trifluoromethoxy) phenylhydrazone; ns, nonsignificant.

Given the possibility that different methods of euthanasia may impact mitochondrial activity, it is important to evaluate whether euthanasia methods can influence OCR values in the murine cornea. We performed the mitochondrial stress assay on corneal biopsy punches in 1.5-mm diameter from male C57BL/6J mice (8-week-old) euthanized either by carbon dioxide asphyxiation or overdosed anesthetic drugs. The results showed no significant difference in basal respiration, maximal respiration, and spare respiration capacities in corneal biopsies between the two euthanasia methods (Figs. 3A–D), suggesting that both euthanasia methods are suitable for this assay.

### Relationship Between Corneal Punch Size and OCR Values

**Figure 4.**
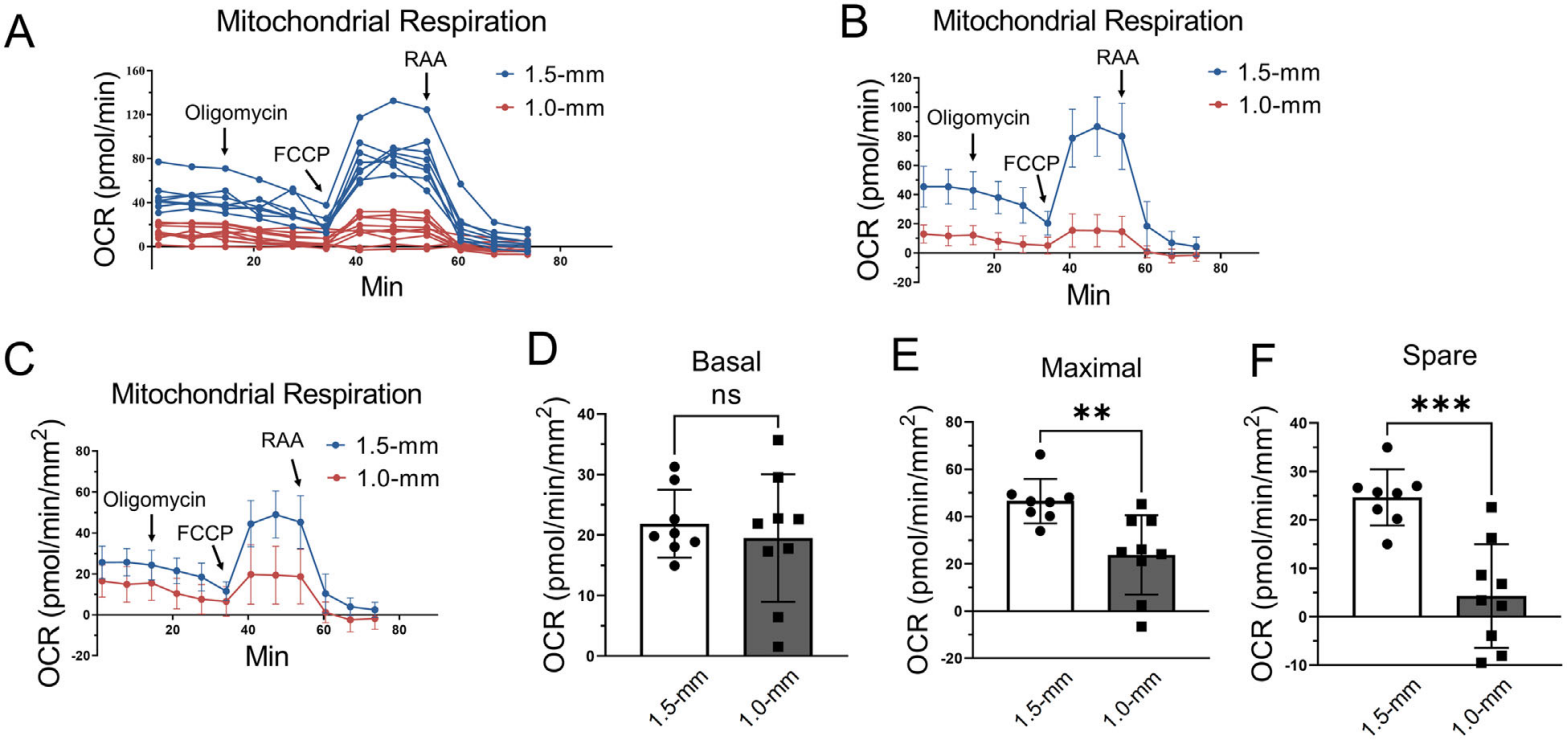
The impact of punch sizes on mouse corneal oxygen consumption. (**A**) Plot showing OCR curves of individual corneal punches in 1.0-mm (*red*) and 1.5-mm (*blue*) diameter. (**B**, **C**) Mitochondrial stress test using corneal punches of 1.0-mm diameter (*n* = 10 corneal biopsies from 5 mice) and 1.5-mm diameter (*n* = 8 corneal biopsies from 4 mice) before normalization (**B**) and after normalization by the area of punches (**C**). (**D**–**F**) OCR values in the corneal punches of 1.0-mm diameter (*n* = 10 corneal biopsies from 5 mice) and 1.5-mm diameter (*n* = 8 corneal biopsies from 4 mice) after normalization by the area of punches. Values are expressed as mean ± standard deviation. ns, nonsignificant. **P < 0.01; ***P < 0.001. FCCP, carbonyl cyanide 4-(trifluoromethoxy) phenylhydrazone.

Agilent recommends OCR values between 20 to 200 pmol/min before normalization for reliable measurements.^14^ To optimize punch size to yield the optimal OCR value, we performed a mitochondrial stress assay on corneal biopsies with the size of 1.0-mm (size 1.0-mm) and 1.5-mm (size 1.5-mm) in diameter. Corneas were dissected from 8-week-old male C57BL/6J mice euthanized by carbon dioxide asphyxiation. Size 1.0-mm yielded lower OCR recording curves, which were below the recommended range, and some biopsies showed poor/flat OCR curves (Fig. 4A). The OCR value from size 1.5-mm started from 20 pmol/min and reached the peak of 140 pmol/min after FCCP injection, which was in the optimal range as Agilent recommended (Figs. 4A and B). After normalization by the biopsy area, size 1.0-mm showed significantly lower maximal and spare respiration capacity, and higher variabilities relative to size 1.5-mm. However, there was no significant difference in basal respiration between size 1.0-mm and size 1.5-mm after normalization by the area (Figs. 4C–F). Some size 1.0-mm biopsies showed negative values on maximal respiration and spare respiration capacity, suggesting possible mechanical damage and decreased viable cells in tissue biopsies, causing variability for analysis. Thus, mouse corneal biopsy punches in 1.5-mm diameter were more appropriate for mitochondrial activity assays than those in 1.0-mm diameter.

### Relationship Between Biopsy Orientation and Corneal OCR Values

**Figure 5.**
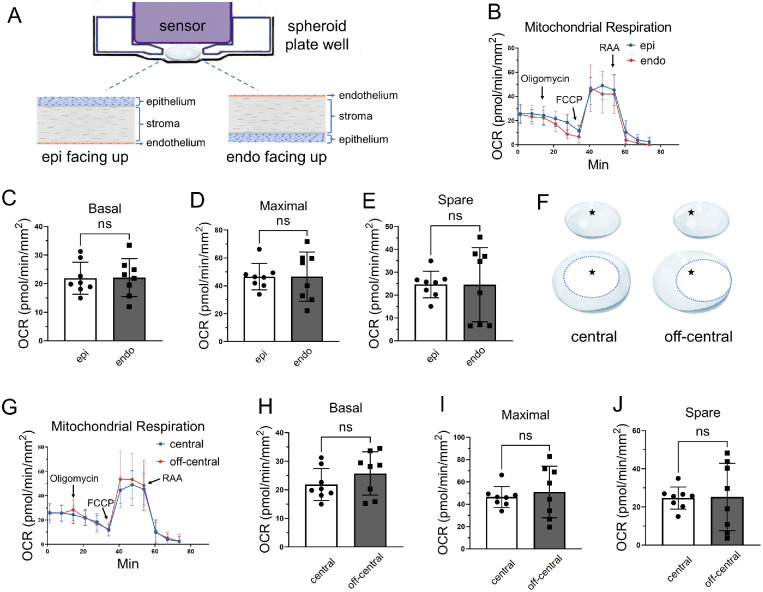
The impact of biopsy orientation and biopsy locations on mouse corneal oxygen consumption. (**A**) A representation figure of the orientation of corneal biopsies with the epithelium (epi) and endothelium (endo) facing the cartridge sensor. (**B**–**E**) The OCR values in the murine corneal biopsy with the epithelial layer (epi) and endothelium (endo) facing upward (*n* = 8 corneal biopsies from 4 mice). (**F**) A diagram showing the location of biopsies in the central or off-central regions of the cornea. A star symbol (★) represents the center of the cornea. (**G**–**J**) The mitochondrial OCR in the punches from the central or off-central areas of the corneas. Values are expressed as mean ± standard deviation, *n* = 8 corneal biopsies from 4 mice. FCCP, carbonyl cyanide 4-(trifluoromethoxy) phenylhydrazone; ns, nonsignificant.

To assess whether the orientation of corneal biopsies in the assay results in different OCR values, we performed a mitochondrial stress assay on corneal biopsies (size 1.5-mm) with either the epithelium side up or the endothelium side up (facing the cartridge sensor). The results demonstrated no significant difference in the basal, maximal, and spare OCR values between the wells with the epithelial layer facing upward and those with the endothelium side up (Figs. 5A–D).

### Relationship Between Biopsy Punch Location and Corneal OCR Values

Considering that corneal epithelial cells in the center are more mature than those in the periphery, it is intriguing to elucidate the impact of biopsy location in the cornea on OCR values. We used size 1.5-mm punches from the central and off-central areas (without limbus) of the cornea from 8-week-old male C57BL/6J mice euthanized by carbon dioxide asphyxiation. The results (Figs. 5E–H) showed no significant difference in mitochondrial activity between the punches from the corneal central and off-central (without limbus) areas, which suggested that off-central biopsy punches did not significantly alter the OCR values.

### The Impact of Sex, Strain, and Age of Mice on Corneal OCR Values

**Figure 6.**
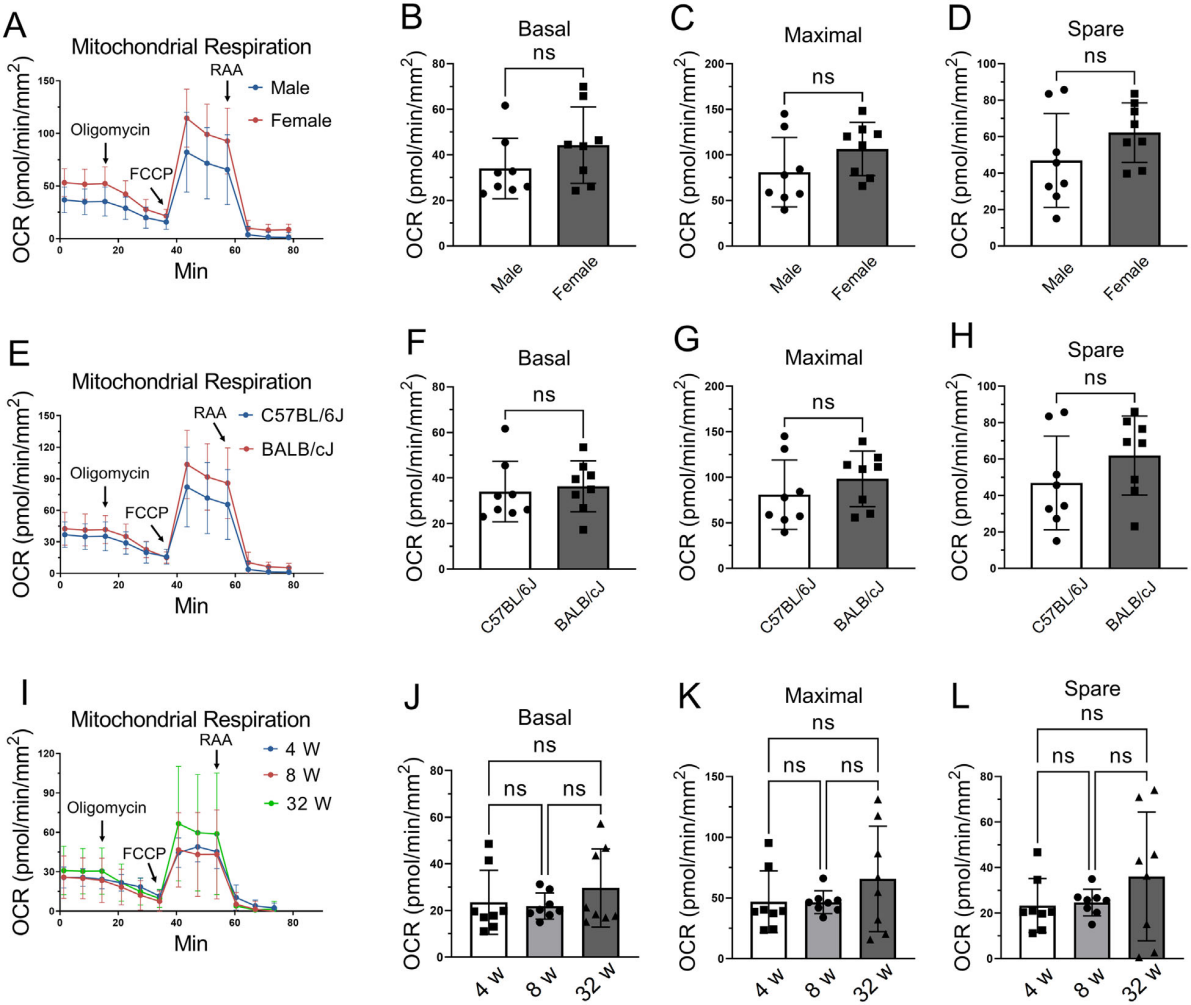
The impacts of sex, strain, and age on murine corneal OCR values. (**A**–**D**) Mitochondrial stress test in the corneal punches from male and female C57BL/6J mice (*n* = 8 corneal biopsies from 4 mice). (**E**–**H**) OCR levels in the cornea punches from C57BL/6J and BALB/cJ mice (*n* = 8 corneal biopsies from 4 mice). (**I**–**L**) Mitochondrial stress test in the corneas from 4-, 8- and 32-week-old male C57BL/6J mice (*n* = 8 corneal biopsies from 4 mice). Values are expressed as mean ± standard deviation. FCCP, carbonyl cyanide 4-(trifluoromethoxy) phenylhydrazone; ns, nonsignificant.

To investigate if there is sex dimorphism in mitochondrial metabolism, we tested the real-time mitochondrial respiration using the size 1.5-mm cornea punches with the epithelium side up from 8-week-old C57BL/6J mice. Although female corneas showed a trend of higher oxygen consumption than males, there was no significant difference in OCR levels between genders as shown by an unpaired Student *t*-test (Figs. 6A–D).

To study the mouse strain difference in corneal mitochondrial metabolism between pigmented C57BL/6J and albino BALB/cJ mice, we compared the real-time mitochondrial respiration using central corneal punches (size 1.5-mm) with the epithelium side up from 8-week-old male mice. Figures 6E–H showed no statistically significant difference in OCR levels between C57BL/6J and BALB/cJ mice.

To compare the corneal mitochondrial function in different ages, we measured the OCR values in central corneal biopsies (size 1.5-mm) with the epithelial side up from 4-, 8-, and 32-week-old male C57BL/6J mice. The results showed no significant differences in corneal OCR levels among these ages (Figs. 6I–L).

### Mitochondrial Respiration in Wounded Corneas

**Figure 7.**
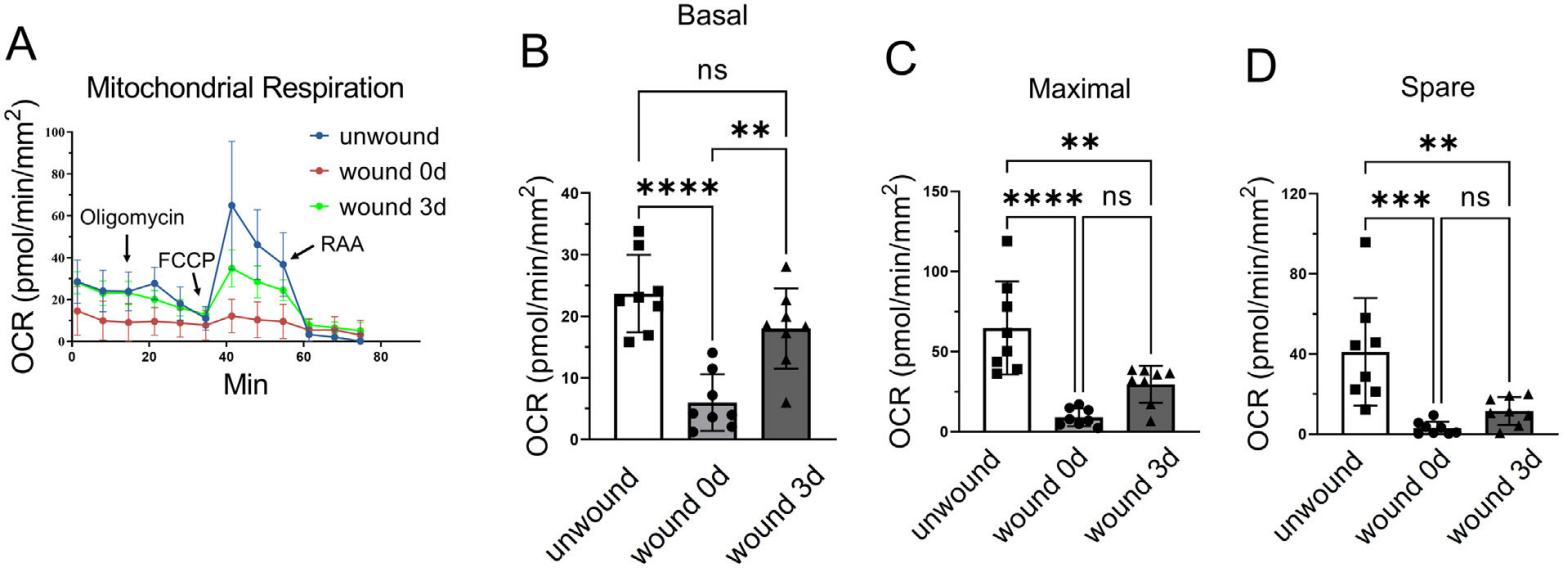
Mitochondrial respiration measurement in wounded corneas. (**A**–**D**) Mitochondrial stress test in the unwounded cornea and corneas at 0 d and 3 d after wound (*n* = 8 corneal biopsies from 4 mice). Values are expressed as mean ± standard deviation. FCCP, carbonyl cyanide 4-(trifluoromethoxy) phenylhydrazone; ns, nonsignificant; **P < 0.01; ***P < 0.001; ****P < 0.0001.

To track mitochondrial activity in the corneal wound healing process, we generated corneal wounds in 8-week-old male C57BL/6J mice by corneal epithelial debridement. On the day of corneal abrasion (day 0), diminished OCR was detected in the corneal biopsies compared with the unwounded corneas, likely owing to the loss of epithelial cells (Figs. 7A–D). At day 3 after corneal abrasion, there was a partial recovery of the OCR values, suggesting that mitochondrial activity was affected by the wound-healing process in the cornea.

## Discussion

Glycolysis and mitochondrial oxidative phosphorylation are two major sources of ATP production in tissues and cells. Therefore, to assess corneal metabolism more precisely, it is necessary to measure mitochondrial activity and glycolysis in fresh corneal biopsies. In this study, we developed and characterized a method to analyze the metabolic profile and mitochondrial function in ex vivo fresh murine corneal biopsy using a Seahorse extracellular flux analyzer. Although previous reports have demonstrated the use of ex vivo retinal biopsies for Seahorse analysis,^15^^–^^17^ to our knowledge, there has been no prior report characterizing real-time metabolism analysis using ex vivo corneal biopsies. Here, we found that in the ex vivo cornea biopsies, mitochondrial oxidative phosphorylation is the predominant source of ATP production. Using this novel method, we discovered that diabetes impaired mitochondrial function in the corneas of two type 1 diabetic mouse models (streptozotocin-induced diabetic and Akita mice), as well as a type 2 diabetic mouse model (*db/db* mice).^12^ This observation emphasized the importance of mitochondrial functional study using fresh murine corneal biopsies. This technique could also assess mitochondrial function in the corneas of humans and other animal models. However, the optimal conditions (such as biopsy size and compound concentration) for applying this method to other species need to be further characterized.

Several studies showed that the metabolic profiles of different organs and tissues might be influenced by different anesthesia or euthanasia methods.^18^^,^^19^ In the present study, for the first time, we compared corneal mitochondrial activity following two commonly used euthanasia methods, an overdose of ketamine and xylazine, as well as carbon dioxide asphyxiation. Our data demonstrated no significant differences in corneal OCR values between these two euthanasia methods, indicating that both euthanasia methods can be used for this assay.

The thickness of the murine cornea is ideal to fit within the 0.25-mm deep circular flat detent in each well of the Seahorse 96-well spheroid microplate.^20^ The detents allowed us to confine the corneal biopsy punches in the center of each well during the assay without using a tissue/cell adhesive such as Cell-Tak. In studies of the retina, 1.0-mm diameter punches were widely used for real-time measurement of OCR.^15^^–^^17^ However, we observed a suppressed and variable maximal and spare OCR in corneal punches of 1.0 mm in diameter, falling below the recommended range. In contrast, biopsies of 1.5 mm in diameter generated higher and more reproducible OCR readings within the range recommended by the manufacturer. After normalization by the punch area, the maximal and spare OCR values from size 1.0-mm biopsies were still lower than those from size 1.5-mm biopsies. Further, some 1.0-mm punches generated negative OCR values, which resulted in greater variability in OCR relative to 1.5-mm biopsies. Preparation of a 1.0-mm biopsy may cause tears in the corneal tissue during the punch operation, which could introduce tissue damage that causes loss of mitochondrial activity. Moreover, corneal punches of 1.0-mm in diameter may be curling and result in tissue movement during the assay, which interferes with OCR readings. Therefore, we recommend 1.5-mm in diameter biopsies for the mouse corneal OCR evaluation.

Strain differences in metabolism have been reported in other tissues.^21^^–^^24^ For example, BALB/cJ mice are known to have a substantially faster visual cycle, suggesting a higher metabolic rate in the retina and RPE relative to C57BL/6J mice.^25^ In addition, C57BL/6J mice have more severe ischemia-induced retinal neovascularization than BALB/cJ mice.^26^ Therefore, the genetic background of mouse models, especially in genetically modified mice, should be carefully considered. Here, we compared corneal mitochondrial activities in these two mouse strains. Unlike the retina, however, we found that BALB/cJ and C57BL/6J mice have similar OCR in corneal biopsies, suggesting no major strain difference in corneal mitochondrial activities, at least between these examined mouse strains.

Age and sex are two other important factors that affect metabolism.^27^^,^^28^ For example, the mitochondrial content and density, mitochondrial DNA copy number, and mitochondrial protein levels decrease with aging in the rodent liver.^29^^,^^30^ Female mice show higher mitochondrial biogenesis in the heart and brain than males.^31^ Female rats possess a higher mitochondrial capacity than males in the liver and brown adipose tissue.^32^^,^^33^ Therefore, we assessed the possible impacts of sex and age on corneal mitochondrial activities. Although a trend of higher OCR was observed in female mice, our results demonstrated no statistically significant difference between males and females in OCR values, at least under physiological conditions. Similarly, we did not observe any significant difference among mice at ages 4, 8, and 32 weeks under physiological conditions. However, whether there is a gender or age difference under disease or stress conditions remains to be studied.

Corneal diseases such as diabetic keratopathy, Fuchs' endothelial corneal dystrophy and keratoconus have been reported to be associated with disrupted metabolic processes, mitochondrial dysfunction, and decreased ATP production.^34^^–^^36^ Mitochondrial stress assays can provide valuable insight into the underlying pathophysiology of these conditions. Several mouse models have been developed to study corneal diseases, such as those with mutations in the transcription factor 4 (*TCF4*) gene for Fuchs' endothelial corneal dystrophy and mutations in the visual system homeobox 1 (*VSX1*) gene for keratoconus.^37^^,^^38^ By measuring corneal OCR in these mouse models, we may be able to better understand the mitochondrial dysfunction underlying these diseases, potentially identification of new therapeutic targets for the treatment of these conditions.

Although assessing mitochondrial function in fresh corneal tissue has advantages over the measurement using cultured cells, this method has its limitations as well. The whole corneal biopsy punch may not be amenable to identifying which layers or cell types have altered mitochondrial respiration. To measure the metabolic activities in the isolated corneal stroma, we scratched off the epithelial layer and/or endothelial layer before the Seahorse analysis. The bare stroma showed a very low OCR value (Fig. 7). This result could be ascribed to mechanical damage to the stroma during the operation, leading to diminished mitochondrial respiration. It may also reflect that the stroma contains a very low cell density, and stromal cells primarily use glycolysis to generate ATP.^12^ The variability of OCR levels on separated corneal layers was relatively large, and a careful and consistent procedure for punching the cornea is needed.

Corneal epithelium wound induced by debridement is a commonly used model for corneal wound healing studies. Our study using corneal biopsy punches taken from different time points after corneal epithelial debridement revealed that trauma and wound healing alter mitochondrial respiration in the cornea. Specifically, corneal epithelial wound decreased OCR values in the corneal biopsies compared to unwounded corneas. Despite the low OCR values observed in 1.5-mm corneal biopsies at day 0 of the wound before normalization, which fell below the manufacturer's recommended range, it seems that this factor did not impact the sensitivity of the readings. This result is evident from the fact that all the basal OCR, maximal OCR, and spare respiration OCR values were positive, unlike the 1.0-mm biopsies. These findings further support the use of 1.5-mm biopsies for this assay. Recovery of the OCR values was associated with corneal wound healing, which suggests that mitochondrial function is required for the maintenance of physiopathological activity in the cornea. Low OCR values at day 0 of the wound are likely due to the loss of epithelial cells. Previous studies showed the corneal wounds were completely healed, which was confirmed by sodium fluorescein staining, in this model on day 3 after wounding.^39^^–^^44^ Surprisingly, the OCR value on day 3 was still significantly lower than the unwounded cornea. This lower OCR may be explained by the fact that the cell number may not be completely recovered to the unwounded level at day 3. It is also possible that proliferating epithelial cells in the wounded area may use glycolysis rather than mitochondrial oxidative phosphorylation. Future studies will be required to measure glycolysis in the wounded area and to elucidate the cause of the changes in mitochondrial functions in the newly healed cornea. Furthermore, some chemicals, proteins, and drugs have been discovered to have therapeutic benefits in the context of corneal wound healing and fibrosis.^45^^–^^49^ It is of great interest to investigate whether these therapeutic effects are associated with corneal mitochondrial function and ATP production.

To obtain reproducible OCR measurements, we normalized the OCR values by the punch area and kept the biopsy punch size consistent. This is a well-accepted method to minimize the need for secondary normalization. However, in certain situations, such as corneal epithelial wounds, an external normalization factor may be necessary based on cell counts, total protein concentration, or genomic DNA copy numbers.

In summary, we optimized a method for examining mitochondrial respiration in fresh ex vivo murine cornea. The findings provided an important tool for researchers seeking to study the mitochondrial function of the cornea.
